# Epistemic and Non-epistemic Values in Earthquake Engineering

**DOI:** 10.1007/s11948-023-00438-0

**Published:** 2023-05-02

**Authors:** Luca Zanetti, Daniele Chiffi, Lorenza Petrini

**Affiliations:** 1 Linguistics & Philosophy Center - L&PIC, Scuola Universitaria Superiore IUSS Pavia, Piazza della Vittoria 15, 27100 Pavia, Italy; 2 Department of Architecture and Urban Studies – DAStU, Politecnico di Milano, Campus Leonardo – Via Bonardi 9, 20133 Milan, Italy; 3 Department of Civil and Environmental Engineering – DICA, Politecnico di Milano, Campus Leonardo – Piazza Leonardo da Vinci 32, 20133 Milan, Italy

**Keywords:** Earthquake engineering, Values, Philosophy of engineering, Models, Uncertainty

## Abstract

The importance of epistemic values in science is universally recognized, whereas the role of non-epistemic values is sometimes considered disputable. It has often been argued that non-epistemic values are more relevant in applied sciences, where the goals are often practical and not merely scientific. In this paper, we present a case study concerning earthquake engineering. So far, the philosophical literature has considered various branches of engineering, but very rarely earthquake engineering. We claim that the assessment of seismic hazard models is sensitive to both epistemic and non-epistemic values. In particular, we argue that the selection and evaluation of these models are justified by epistemic values, even if they may be contingently influenced by non-epistemic values. By contrast, the aggregation of different models into an ensemble is justified by non-epistemic values, even if epistemic values may play an instrumental role in the attainment of these non-epistemic values. A careful consideration of the different epistemic and non-epistemic values at play in the choice of seismic hazard models is thus practically important when alternative models are available and there is uncertainty in the scientific community about which model should be used.

## Introduction

Earthquake engineering, unlike other types of engineering, has not received a proper philosophical analysis regarding its foundations, methods, and applications. The goal of this paper is to provide the first philosophical account (to our knowledge) of the current practice of *ensemble modeling* in earthquake engineering from a value-based perspective.

Competing seismic models are typically available. These models often provide largely different estimates of the frequency of certain events (for example, earthquakes with a specific magnitude) and therefore these models produce different estimates of seismic hazard. However, it is often impossible to determine which model is correct due to a shortage of historical data in conjunction with other factors such as modeling and epistemic uncertainties. Instead, ensembles of different models are often used to quantify seismic hazards.

Our main claim will be that ensemble modeling in earthquake engineering is guided both by *epistemic values*, which are related to knowledge, and by *non-epistemic values*, which are related to practical aims and goals. More specifically, we claim that the *selection* of the models that are included in the ensemble and the *evaluation* of those models by panels of experts are both justified by epistemic values. By contrast, the *aggregation* of those models into the ensemble itself is justified by its compliance with some non-epistemic values. The explicit considerations of the different epistemic and non-epistemic values at play are practically important when different seismic hazard models and different methods to provide an assessment of those models are available and the analyst must determine which method is the most appropriate in a particular circumstance.

We will proceed as follows. We will first introduce the distinction between epistemic and non-epistemic values. The interplay between these values will be discussed by referring to the risk analysis that was conducted in the Fukushima region years before the well-known nuclear disaster. We will then present a general framework for the assessment of seismic hazard models (the Selection, Evaluation, and Aggregation framework - SEA) that accounts for how probabilistic seismic hazard analysis (PSHA) is currently practiced. We will claim that the selection and the evaluation of seismic hazard models is justified by epistemic values. We will also argue that the the aggregation of these models into ensembles is justified by non-epistemic values (in particular, model ensembles enable specific types of decisions that would not be available if only one model was considered). Finally, we will formulate some closing remarks.

## Epistemic and Non-epistemic Values in Science and Engineering

The distinction between epistemic and non-epistemic values is familiar from the philosophy of science (Kuhn, 1977; Douglas, 2000; Dorato, 2004; Lacey, 2005; Steel, 2010; Elliott & McKaughan, 2014; Ward, 2021; Elliott, 2022).

Epistemic values are related to the pursuit of knowledge. They include for instance truth and truth-likeness, objectivity, error reduction, simplicity, effectiveness, elegance, fruitfulness, scope, accuracy, robustness, predictive power, novelty, applicability, ontological homogeneity, and explanatory power.

By contrast, non-epistemic values are related to practical aims and goals. They include for instance ethical values (safety, beneficence, non-maleficence, autonomy), political values (sustainability, equality, justice), and economic values (feasibility, profit).

However, some values are neither clearly epistemic nor clearly non-epistemic (McMullin, 1982). Therefore, in some concrete cases, it may be better to say that values can have epistemic and non-epistemic *facets* that may sometimes be hard to disentangle. Even so, distinguishing epistemic and non-epistemic values may be still conceptually fruitful (Chiffi, 2021). An example of a value for which it can be difficult to be recognized as fully epistemic or fully non-epistemic in earthquake engineering is *scientific responsibility*. In the influential report of the US Senior Seismic Hazard Analysis Committee (SSHAC, 1997) scientific responsibility is defined as “the responsibility not only for the accuracy and completeness of the results but also for the process used to arrive at the results” (p. 25). According to the SSHAC report, scientific responsibility encompasses both integrity, which requires the scientist to exercise their best professional judgment, and diligence, which requires that the scientists “learn about the most recent advances in the field, often by direct contact with other experts” (p. 28). Therefore, scientific responsibility has both epistemic aspects (related to the accuracy of the model) and non-epistemic aspects (integrity and diligence) that are instrumental in the attainment of the epistemic goals of the scientist.

The importance of epistemic values to science is universally recognized, while the role of non-epistemic values is sometimes considered disputable. More precisely, it is hard to deny that scientific practice has been guided in the past, and sometimes still is guided, by non-epistemic values, for example, political and economic values. What is controversial is whether the scientific inquiry should be guided and justified by such values (Rooney 1992). Here we can distinguish between *denialists* and *compatibilists*. Denialists claim that science should be free from non-epistemic value. They often distinguish between the contextual role of values (contingently associated with scientific activity) and the constitutive role of values (necessary to the scientific enterprise) and argue that even though non-epistemic values can have contextual importance, they are not constitutive of scientific practice. By contrast, compatibilists claim that non-epistemic values play a role in science. They sometimes stress the difference between *pure science* and *applied* science. On the one hand, it is natural to think that in the case of pure sciences, the intrinsic values (i.e., the ultimate aims of scientific inquiry) are all epistemic, even if non-epistemic values, though not intrinsic to science, may have nonetheless an instrumental role in the attainment of some constitutive (scientific) goal. On the other hand, non-epistemic values are arguably more relevant in the case of applied sciences, where the goals are often practical and not merely scientific.

For example, the goal of *engineering* is often to solve a particular problem based on the available science, methods, and techniques rather than just to increase knowledge. For this reason, we can expect that non-epistemic values will be more important to engineering than to science. A general analysis of the role of non-epistemic values in engineering is provided by Diekmann and Peterson (2013). Diekmann and Peterson argue that the formulation of models in engineering is influenced by non-epistemic values. One of their examples is the calculation of parameters that are used as safety criteria. In this case, the model includes only those parameters that are considered relevant for safety. According to Diekmann and Peterson, “safety, which is a paradigmatic example of a non-epistemic value, influences the choice of represented parameters” (p. 212). Non-epistemic values may also counterbalance epistemic ones (for example, the usability of a model can outscore its accuracy in representing the target system) and influence the choice of models.

Diekmann and Peterson also argue that non-epistemic values are constitutive to the practice of engineering. They make a normative claim: engineering is not only influenced by but also ought to be influenced by non-epistemic values. Their argument is as follows. They claim that (i) engineering models ought to be developed with some specific goal in mind (i.e., solving a particular problem) and sometimes (ii) these goals ought to be non-epistemic (e.g., safety). They add that (iii) the influence of epistemic and non-epistemic values determines whether the model satisfies the relevant goals. From this, they conclude that (iv) some models ought to be influenced by non-epistemic values.

So far, the philosophical literature has considered various types of engineering (see Donovan, 2012; Bokulich & Oreskes, 2017), but very rarely earthquake engineering. In this paper, we show that earthquake engineering is an intriguing example of interaction between epistemic values and non-epistemic values in virtue of their impact on different forms of uncertainty. Our claim will mainly be descriptive: earthquake engineering is in fact influenced both by epistemic values and by non-epistemic values. We will also discuss whether acknowledgment of this point should change how earthquake engineering is practiced. In the next section, we will start by considering a motivating example.

## Epistemic and Non-epistemic Values in Earthquake Engineering

Decisions to reduce seismic risk imply a coherent methodology to assess the consequences of future earthquakes (and their level of uncertainty) on people and structures (McGuire, 2004). This means that uncertainty and value-based considerations on the effects of earthquakes characterize this kind of risk. More specifically, non-epistemic values (e.g., economic, ethical, or political ones) intended to reduce the impact of the earthquake on people and structures are intimately connected with the engineering methodology of seismic risk mitigation. Of course, also epistemic values regarding for instance the reliability of information about where earthquakes can originate, or what ground-motion intensity they can produce, are essential to deal with seismic risks. These two kinds of values are intimately connected and establishing some thresholds among them is usually required when taking engineering-based decisions (Van de Poel, 2009).

A particularly clear example of the interplay between epistemic and non-epistemic values in earthquake engineering is the hazard analysis of potential earthquakes produced for the Fukushima power plant before the nuclear disaster in 2011. On March 11, 2011, an earthquake of magnitude 9.0 occurring 130 km offshore of Japan produced a tsunami approximately 13 m high. When the tsunami hit the coast, the nuclear power plant in Fukushima was inundated. In the following days, and after multiple engineering failures, two of the three reactors that were operational at the time of the incident exploded, causing another one to explode a day later. This last reactor contained spent fuel rods, whose exposure caused a large-scale emission of radiation. The Fukushima incident was one of the major nuclear disasters that ever occurred.

When the nuclear plant was built between 1967 and 1971, a seismic risk analysis was performed to determine the maximum earthquake that could strike the region of Fukushima for the chosen return period (approx. 10^5^ years). However, the risk analysis that preceded the construction of the Fukushima power plant was based on a limited historical catalog. In particular, the maximum earthquake considered was the Shioya-Oki earthquake in 1938, which had a magnitude of 7.8. It was therefore estimated that a tsunami caused by a seismic event in the region may be at most 6.1 m high, underestimating the true value of more than a half.

Notably, a second power plant, in Onagawa, was also hit by the tsunami. But despite the Onagawa plant being closer to the epicenter than Fukushima, this second plant did not fail due to the tsunami. This was because of a different risk analysis that considered a wider timeframe which also included an earthquake that occurred in 869 with an estimated magnitude of 8.3, then concluding that an earthquake-generated tsunami may be up to 13.6 m high. So, the Onagawa power plant was substantially safer than the Fukushima one. As stressed by Taebi (2020, p. 31), “the design of the Fukushima Daiichi plant seems to have taken too short a historical period into account”.

We can see that purely epistemic considerations had a role in the quantification of seismic and tsunami hazards for the Fukushima power plant. The risk analysis was based on available data and took into consideration, different models. However, non-epistemic considerations played an even more important role. In particular, the risk assessment produced by the company that runs the Fukushima power plant had only “a vague reference to investigation reports by research institutes”, but ignored models that suggested higher estimates (Synolakis & Kânoğlu, 2015, p. 9). As we saw, a very different analysis was considered for the Onagawa power plant which suggested that the risk may have been much higher than what was originally considered when the Fukushima power plant was built. Some have suggested that this difference is explained also by non-scientific factors. In particular, “one official suggested that because decision-making for the Ongawa nuclear plant project at Tohoku Electric Power Company involved local personnel, top management there may have been more receptive to making costly siting changes” (Acton & Hibbs, 2012, p. 30), even though the company’s failure to take into considerations new scientific insights may have also been due to inefficient decision-making (Acton & Hibbs, 2012; Synolakis & Kânoğlu, 2015).

The Fukushima accident suggests that earthquake engineering may be sensitive to both epistemic values, such as accuracy and fit with historical data, and to non-epistemic values, such as safety and economic factors. But how are these values related in practice? We will now discuss a concrete example concerning the assessment of hazards. We will first briefly introduce probabilistic seismic hazard analysis and distinguish between different phases in the assessment of seismic hazard models. Our discussion will show clearly that different phases are justified by different types of values.

## Selection and Evaluation in Probabilistic Seismic Hazard Analysis (PSHA)

Probabilistic Seismic Hazard Analysis (PSHA) has become common practice in earthquake engineering.^1^ PSHA (cf. Baker et al., 2021 for an overview) estimates the seismic hazard at a site as the probability that a specific ground-motion intensity level is exceeded at that site in a specified period of time (for example, a frequency of 10% in 50 years, corresponding to a return period of 475 years).^2^

A probabilistic seismic hazard model consists of three main parts. The first one is the earthquake rate model (ERM). An ERM of the area in which the site of interest is located consists of a set of events that may affect the site characterized by their position in the area, their magnitude, and their frequency. The position is usually represented as a point on a plane. The second component is a ground-motion model (GMM), or ground-motion attenuation relation. A GMM expresses the ground motion determined by an event at a given source as a function of the magnitude of that event at its source and the distance between the source and the site. The third and final part consists in the integration over all relevant distances and magnitudes that determines the probability that each specific ground-motion intensity level *a* is exceeded. If the site can be affected by *n* sources, the overall seismic hazard corresponds to the combination of the effects of all possible seismic events weighted by their frequency.

The quantification of seismic hazards is subject however to two types of uncertainty (Zanetti et al., 2023). First, there are *aleatoric* uncertainties, that are due to the stochastic—rather than deterministic—character of seismogenic events. For example, it is uncertain where future earthquakes will occur (spatial uncertainty), when they will occur (temporal uncertainty), and which level of ground motion they will produce (ground-motion uncertainty). Second, there are *epistemic* uncertainties, that are due to insufficient data and/or incomplete knowledge of the phenomena. We can further distinguish within epistemic uncertainties between *model* uncertainty and *parametric* uncertainty. Model uncertainty concerns the general form of the equation. For example, it can be uncertain whether the magnitude distribution follows the Gutenberg-Richter equation.^3^ Parametric uncertainty, by contrast, concerns the value of the parameters of the models. For example, one can be uncertain about the *a*-value and the *b*-value in that same equation for the zone of interest.

Epistemic uncertainties are usually included in the calculation of seismic hazards using logic trees (Kulkarni et al., 1984). Setting up a logic tree involves two steps. First, different seismic models are selected together with alternative estimates of the values of their parameters. Second, a *weight* is assigned to each branch that departs from a node in the tree.

The nodes of the tree correspond to a choice between alternative models or alternative evaluations of the parameters. A complete branch of the tree corresponds to a complete seismic hazard model that estimates the frequency of exceedance corresponding to each ground-motion intensity and, therefore, computes a hazard curve.^4^

The weights assigned to the models are expressed as probabilities. Informally, the weight assigned to a branch departing from a node is the probability that the choice of values corresponding to that node is correct given that everything proceeding that node is correct. The overall epistemic uncertainty corresponds to a bundle or family of hazard curves, whose spread corresponds to the variance in the estimates of the frequency of an event with a specific return period produced by the models included in the logic tree.

We shall now propose a general framework that describes the current practice of PSHA. We call it the *Selection, Evaluation and Aggregation Framework* (SEA).^5^ This framework is divided into three phases.

The *selection* of the models that are included in the logic trees is usually based on a literature review. The selection aims to collect all the models that have been published in the literature and that can be applied to the site of interest. More recently, some PSHA studies have also included models that have been solicited directly by the responsible for the study through a call for contributions (Meletti et al., 2021).

The *evaluation* of the models is performed by a panel of experts and consists of the assignment of a weight to each model. According to the procedure detailed in the influential report of the US Senior Seismic Hazard Analysis Committee (SSHAC), each expert provides an “individual judgment” on the credibility of the models (SSHAC, 1997). Experts may also agree on a “community distribution” that represents the overall judgment of the members of the panel (NCR, 1997; Budnitz et al., 1998). In this case, the goal of the evaluation is also to reach a consensus among experts. In particular, all experts should agree that a particular composite probability distribution represents them as a group, or, more weakly, that all experts agree that distribution represents the overall scientific community, rather than, more strongly, agreeing that a specific value or model is correct.

This weak type of consensus can be achieved in five different ways. First and foremost, (1) experts might explicitly agree on a particular probability distribution. If not, then their judgments can be integrated either (2) by assigning the same weight to each judgment, or (3) by assigning unequal weights. In this last case, one can either (4) assign a quantitative weight or (5) perform a qualitative evaluation without assigning a numerical value to the judgments. This last option is considered the less desirable possibility (SSHAC, 1997; NCR, 1997; Budnitz et al., 1998).

Finally, the *aggregation* of models into an ensemble consists in producing a logic tree with the selected models and with the weights based on the judgments provided by the experts. This aggregation is performed either by a “Technical Integrator” (TI) or by a “Technical Facilitator/Integrator” (TFI). The TI proposes a probability distribution that represents the judgments expressed by the experts. The TFI encourages the interaction between the experts to reach a consensus and assembles the logic tree with the weights given by the panel. In this last case, the responsibility for the result is shared between the experts and the TFI (SSHAC, 1997, p. 31). In this section, we discuss the selection and evaluation of seismic hazard models. We will consider the aggregation in "Aggregation in PSHA and non-epistemic values" section below.

In the philosophy of science, it is common to distinguish the *context of discovery*, which comprises the actual circumstances in which a scientific result is achieved, from the *context of justification*, which consists of how that result is established by an experiment and in relation to the body of scientific evidence and knowledge. A specific value is constitutive of scientific practice if it provides the justification (based on methods and reasons) and not merely the discovery of new results. It has often been claimed that, at least in pure sciences, non-epistemic values may play a role in the discovery but have no constitutive role. We will now show that in the assessment of seismic hazard models, epistemic values only justify the first two of those phases (*selection* and *evaluation*), even if they may be contingently influenced by non-epistemic values, whereas the third and last phase (*aggregation*) is mainly justified by non-epistemic, even if epistemic values may also play an instrumental role. This distinguishes earthquake engineering, and engineering in general, from pure science since non-epistemic values seem to play a constitutive role not only in the discovery but also in the justification.

The selection of the models seems clearly to be justified by epistemic values. The goal of the selection is indeed to include in the logic tree all the published models that can be applied to the site of interest and to prevent the analyst from considering only the models with which he or she is more familiar with. The inclusion of the models in the logic tree is often motivated by the comparability between the site for which the model was originally developed and the site of interest in terms of their geophysical properties. The similarity between the two sites makes it likely that the model describes the ground motion at the site (Bommer et al., 2010; Cotton et al., 2006).

The evaluation of seismic models is mainly justified by epistemic values as well. The starting point of the evaluation is usually a dataset of ground-motion recordings from the area in which the models should be applied. The experts should be able to justify their judgments based on those data (Klügel, 2011), together with considerations about the assumptions and the logic of the models. Indeed, as stressed for instance by Bommer et al. (2010), “a model may also be rejected by the analyst if it does not include the influence of a factor known to exert a marked influence on the ground motion, for which a range of values or classes is present in the underlying strong-motion dataset, [for example] faulting” (p. 788). Vice versa, a model may receive a higher weight rather than another model if it contains fewer independent parameters. *Explanatory power* is, in general, an epistemic value.

For example, Scherbaum and Kuen (2011) describe a toy model for the evaluation of different ground-motion equations. Models are evaluated concerning three quality criteria, namely data coverage, belonging to the specific geophysical environment, and type of processing. Each model receives a grade based on their performances, and the grades for each criterion are then normalized to one to obtain the weights. As Scherbaum and Kuen emphasize, “these absolute grades express how well each of the models performs with respect to the sum of all quality criteria applied. Re-normalizing these absolute grades, to sum up to 1 over all models results in values which might seem to be applicable as logic tree weights”. The weights are therefore measures of the *representational accuracy* of the model, which is an epistemic value.

Models can be evaluated also on their forecasting performances with respect to a set of independent data (for example, Backer & Gupta, 2016; Marzocchi & Jordan, 2018; Secanell et al., 2018). These data consist either in historical data that were not considered in the formulation of the models or in new data that have been collected. In the last case, the data consist of yearly recordings of seismic activity, and the models are ranked according to the accuracy of their forecasts. The same model can rank higher than another with respect to historical data, but lower with respect to new data. This may be because historical catalogs contain both small and strong earthquakes, whereas early recordings will likely contain small earthquakes that are more frequent, rather than strong ones that are rarer. *Forecasting accuracy* is indeed another epistemic value.

The view that the evaluation of the model is justified by epistemic values is further supported by considering different interpretations of the weights assigned to the branches of a logic tree.^6^

Logic trees have become almost universally diffused in PSHA, to the point that today it is “very rare to see a published hazard study or a site-specific PSHA that does not include a logic tree” (Bommer & Scherbaumb, 2008, p. 997). Despite this, there seems to be little consensus on what those weights represent.

It is natural to think that the weights correspond to the probability that the model is true or correct. This interpretation is in fact suggested by some scholars in engineering (cp. for example Musson, 2005, Abrahamson and Boomer, 2005). However, this interpretation faces two problems.

First, scientific models are not strictly speaking true or false in the same sense in which scientific statements are (Frigg & Nguyen, 2020). A model may represent some features of the target system, in this case, seismic events, accurately or inaccurately. Second, it is often said hastily that “all models are false” in the sense that each model involves some degree of idealization with respect to the target system. Two models can moreover be *compatible* with each other, if they represent different features of the target system (for example, area-source models, data models, geophysical models, etc.) but they also be *conflicting* with each other, for example, if they generate different estimates of the frequency of the same event of interest.

Finally, historical catalogs of seismic events, which often comprise only a few centuries, are often not sufficient for complete validation of seismic hazard models, which would require thousands of years of data. The frequency of seismic events might therefore be confirmed in principle, but often not in practice. In particular, the experts’ judgments can diverge widely from each other even though they consider the same set of historical data provided as input of the study (SSHAC, 1997). Therefore, weights cannot be interpreted as the probability that the model is correct, and must be understood in some other ways, for instance as follows.

Musson (2012) claims that the weights in a logic tree should be interpreted as the analyst’s “estimate that [the model] is the best model available” (p. 1295). However, the phrase “best model” used by Musson can be interpreted in (at least) two different ways. First, one may say that a model is the best one available because it is the one that is closest to the “true” process of occurrence of the seismic events in the area under consideration. In this case, this second interpretation will be equivalent to the first one, which we have already discussed. Alternatively, a model can be the best one available *with respect to some specific goal of the analyst*. This makes this second interpretation closer to the one that we will now consider.

Scherbaum and Kuehn (2011) claim the correct interpretation of the weights is that they are “subjective estimates for the degree-of-certainty or degree-of-belief … that the corresponding model is the one that should be used” (p. 1238). This interpretation has practical consequences for how the weights are assigned to the models. In particular, as Scherbaum and Kuehn point out, this interpretation does not imply that the weights assigned to the available models sum up to one. Indeed, the analyst may think that none of those models is the model that should be used and decide to develop other models instead.

Scherbaum’s and Kuehn’s interpretation can be usefully compared with the *Adequacy-for-purpose View* of models that has recently been proposed in the philosophy of science (cf. Bokulich & Parker, 2021; Parker, 2020).

According to this view, scientific models are assessed with respect to their adequacy of fitness to a particular purpose, and not merely on whether those models are confirmed by available data. A purpose is a specific goal or aim that one wants to achieve by formulating the model. According to Parker, a model is *adequate-for-purpose* if, in the relevant contexts in which that model is used, the relevant purpose is likely to be achieved.^7^

As emphasized by Bokulich and Parker (2021, p. 31),

Scientific modeling is an activity undertaken by agents with specific goals and purposes in mind, such as the prediction or explanation of a phenomenon. […] models are not just representations, they are tools that are controlled and selected, and manipulated, with an eye on achieving specific epistemic or practical purposes.

As we have seen based on the analysis of how weights are understood in PSHA, the evaluation of the seismic hazard models is usually based on epistemic values. However, in practice, it may *contingently* happen that the selection and evaluation of seismic hazard models are influenced by non-epistemic values, for example, personal connections between the experts, belonging to particular research groups or traditions, and professional prestige. This notwithstanding, the selection of the models that are included in the study should be determined only by their fit with historical data and similarity with the geophysical properties of the site, and the individual judgments expressed by the experts should be grounded on the available historical data and the state-of-art knowledge of the area of interest, rather than on social and pragmatic considerations (Klügel, 2011). In the next section, we will claim that non-epistemic values have the crucial role of justifying the aggregation of the models into ensembles in the third phase of PSHA.^8^

## Aggregation in PSHA and Non-epistemic Values

In this section, we claim that the aggregation of seismic models is in fact motivated by non-epistemic values. The declared aim of the logic tree is to “represent the center, the body, and the range of technical interpretations that the larger informed technical community would have if they were to conduct the study” (SSHAC, 1997, p. 21). The fact that ensemble modeling in PSHA aims to “represent” the judgments of the experts indicates that epistemic values may also be involved: an ensemble of models may be praised because it accurately represents the uncertainty in the scientific community. However, the final goal of the aggregation is *not* to provide a faithful representation of the judgments of the experts, but to provide an assessment of seismic hazard that incorporates the judgments of different experts and that is appropriate for a specific situation or a specific goal. The epistemic value of representing the overall epistemic uncertainty is therefore *instrumental* to attaining a different, non-epistemic goal. We will illustrate this point by discussing an example from the practice of PSHA.

Seismic hazard is typically estimated in either of two ways. First, the seismic hazard can be calculated as the *mean* value produced by the logic tree. Alternatively, the seismic hazard can also be estimated using *percentiles* from the family of hazard curves produced by the logic tree (for example, the 85th percentile or the 90th percentile).

The mean hazard is calculated by the total probability theorem as the average of all the frequencies of exceedance estimated by each curve weighted by the probability assigned to that curve. The use of mean values in the estimation of seismic hazard has mostly been defended by appealing to a probabilistic approach that treats aleatoric and epistemic uncertainties in the same way (McGuire et al., 2005; Musson, 2005). The choice of mean hazard instead of percentiles may be guided by practical considerations as well. McGuire et al. (2005) mention the example of the US Nuclear Regulatory Commission (USNRS) which in 2001 released the *Reactor Safety Goal Policy Statement*. In their document, the USNRS estimates safety criteria for nuclear power reactors using mean frequencies of the occurrence of a meltdown. The decision of the USNRS to use mean frequencies rather than percentiles was motivated by two pragmatic considerations. The first one is that mean values are usually easier to compute than percentiles. The second reason is that these values can be incorporated more easily into a cost–benefit analysis that compares mean economic losses of different options.

In the case of percentiles, one considers as a reference the curve that represents the values that are exceeded according to a specified percentage of the models that are included in the logic tree. As emphasized by Abrahamson and Bommer (2005), “the hazard curve taken as the basis for design should be chosen on the basis of the fractile that reflects the desired degree of confidence that the safety level implied by the selected annual frequency of exceedance (or return period) is being achieved” (p. 607). It is a contextual matter which percentile is the one that should be considered in a given situation because it depends on the level of confidence of the estimate that one wants to achieve with respect to the available models. Decisions based on percentiles take into account the majority of the experts. Therefore, the preference for percentiles in the estimation of seismic hazard seems to be justified by non-epistemic values as well.^9^

The choice of a specific value for seismic hazard (for example, the mean hazard or the 90th percentile) can depend on which non-epistemic goal is pursued. For example, there can be political reasons to choose the mean value of the logic trees. As stated for instance by Marzocchi and Zechar (2011), “one of the main goals of decision-makers is to minimize possible a posteriori critiques if a forecast model fails” (p. 446). Avoiding criticisms is easier if the decision-maker has considered all the available models. The mean hazard calculated by the logic tree is not the prediction of the best model, that is, the prediction of the model with the highest weight, but rather the average of all the predicted values weighted by the probabilities assigned to the experts. Therefore, no single expert may believe that the final estimate is “the one that should be used”, even if the ensemble of models represents the judgments of the scientific community as a whole. So decisions made based on the mean value produced by the ensemble take into account the totality of the scientists.

In some situations, the decision-makers could aim to make decisions that are robust in the sense that the estimate is correct according to a broad range of models but not according to all models. This may be justified because the stakes are particularly low, for example, the construction of a structure with low exposure (e.g. a deposit) in a region with limited seismic activity (that is, very long return periods of critical events) where no human lives would be lost in case of collapse. In low-stakes situations, the decision-makers may ignore extreme models that predict stronger seismic events with higher frequencies. By contrast, if the stakes are extremely high (for example, the construction of a power plant), the decision-maker may consider the highest frequency estimated by the ensemble of models for the event of interest, or even the ‘maximum credible earthquake’ at the site (Krinitzsky, 2002). This strategy can be justified especially if the decision-maker has reasons to think that the scientists are particularly conservative in their projections or that they have a bias toward less alarmistic forecasting (Brysse et al., 2013).

A final point that is worth mentioning is that the way in which uncertainties are represented mathematically can also be influenced by non-epistemic values. The judgments of the experts are often elicited as qualitative judgments about the models (which model should be used) and these judgments are then turned into probabilities (Scherbaum and Kuhen, 2011). The integrators usually have some choice on how they represent the judgments of the experts (Marzocchi & Jordan, 2018). As remarked for example by Parker and Winsberg (2018), “real-world agents, including scientists, decide how to represent these probabilities, often using distributions that are uniform, binomial, Poisson, normal, etc. These decisions, like other methodological decisions in science, can be subject to inductive risk considerations” (p. 127; see also Steel, 2015).

Summing up, the assessment of seismic hazard models is neither a purely scientific nor a purely practical enterprise, but it is sensitive to both epistemic and non-epistemic values. Of the three phases of the assessment, namely selection, evaluation and aggregation, the first two phases are justified by epistemic values, even if non-epistemic values may contingently influence them, whereas the third phase is justified primarily by non-epistemic values, even though epistemic values may play an instrumental role.^10^

## Conclusion

We have discussed some normative elements of earthquake engineering. Starting from the classical distinction between epistemic and non-epistemic values in science and engineering, we critically analysed the role of values in the formulation and validation of models for hazard evaluation in earthquake engineering and we discuss a concrete example. Then, we focused on those models that are probabilistic. We argue that probabilistic seismic hazard analysis (PSHA) is sensitive to a mixture of both epistemic and non-epistemic values. More specifically, on the one hand, the selection and the evaluation of seismic hazard models are both justified by epistemic values such as the accuracy of the model and the fit with historical data. On the other hand, the aggregation of models into an ensemble using logic trees is justified by non-epistemic considerations, and in particular, ensemble models allow potential stakeholders and decision-makers to rely on a hazard assessment that incorporates the judgments of different experts. Finally, being aware of the specific aim to attain and thinking about the epistemic and non-epistemic values consequently involved is important when different models are available and there is uncertainty in the scientific community about which model should be used.

## Footnotes

1 Risk can be characterised as a function of *hazard* (the probability that an unwanted event occurs), *vulnerability* (the probability of suffering a loss if the adverse event occurs) and *exposure* (the magnitude of the eventual loss) (UNISDR, 2015). This paper focuses on seismic hazards; however, similar considerations may apply to the estimation of vulnerability in earthquake engineering.

2 The return period is the converse of the frequency.

3 The Gutenberg-Richter equation, which states that the number of events with magnitude *m* is 10^*a* – *b*m^, where *a* and *b* are values that characterise the seismic zone in which the site is located and expresses the ratio between low-intensity and high-intensity seismic events.

4 A hazard curve plots ground-motion intensities (on the x-axis) and their frequencies of exceedance (on the y-axis). The characteristic shape of the hazard curve displays the fact that earthquakes that produce strong intensities have longer return periods (bottom right of the curve) while earthquakes that produce small intensities have shorter return periods (top left of the curve).

5 Our framework is different from the two-stage (elicitation and integration) framework described in SSHAC (1997) since it distinguishes more clearly between selection and evaluation of the models and can be applied to methods of model evaluation that are not based on the elicitation of judgments from the experts.

6 Recall that each branch of a logic tree corresponds to a model, and that the evaluation of the models is performed by assigning a weight to them.

7 Parker (2020) also distinguishes between different senses of adequacy-for-purpose, namely, as success in a particular instance of use or as reliability of a type of use of the model. Parker’s distinction is not relevant in this context.

8 It is not surprising that risk analysis in general can be influenced by non-epistemic values, since risk also involves vulnerability and exposure (fn. 1). Therefore, it may be reasonable to require that models are more sensitive to catastrophic risks than to ordinary risks. By contrast, hazard analysis aims at estimating an objective quantity, namely the frequency of an adverse natural event, and the role of non-epistemic values is less evident.

9 The choice of a specific percentile is a case in which an epistemic decision is determined by non-epistemic considerations, and it is similar to the case of ‘inductive risk’ concerning Type I/Type II errors in hypothesis testing; see e.g., Brigandt (2015) and Chiffi (2021).

10 In this paper we have not addressed the normative question whether earthquake engineering ought to be influenced also by non-epistemic values or not. At any rate, nothing excludes that there might be purely epistemic reasons for using ensemble models; for example, an anonymous reviewer suggested that ensemble modelling may be justified by a suitable variant of Condorcet’s Jury Theorem.
